# Perturbation of Serotonin Homeostasis during Adulthood Affects Serotonergic Neuronal Circuitry

**DOI:** 10.1523/ENEURO.0376-16.2017

**Published:** 2017-04-11

**Authors:** Marta Pratelli, Sara Migliarini, Barbara Pelosi, Francesco Napolitano, Alessandro Usiello, Massimo Pasqualetti

**Affiliations:** 1 Department of Biology Unit of Cell and Developmental Biology, University of Pisa, Pisa 56127, Italy; 2 Ceinge Biotecnologie Avanzate, Naples 80145, Italy; 3 Department of Molecular Medicine and Medical Biotechnology, University of Naples Federico II, 80131 Naples, Italy; 4 Department of Environmental, Biological and Pharmaceutical Sciences and Technologies, University of Campania, Luigi Vanvitelli, Italy; 5 Center for Neuroscience and Cognitive Systems, Istituto Italiano di Tecnologia, Rovereto, TN 38068, Italy; 6 Neuroscience Institute, National Research Council (CNR), Pisa 56124, Italy

**Keywords:** 5-hydroxytryptophan, serotonergic innervations, serotonin, serotonin homeostasis, tryptophan hydroxylase 2

## Abstract

Growing evidence shows that the neurotransmitter serotonin (5-HT) modulates the fine-tuning of neuron development and the establishment of wiring patterns in the brain. However, whether serotonin is involved in the maintenance of neuronal circuitry in the adult brain remains elusive. Here, we use a *Tph2^fl^°^x^* conditional knockout (cKO) mouse line to assess the impact of serotonin depletion during adulthood on serotonergic system organization. Data show that the density of serotonergic fibers is increased in the hippocampus and decreased in the thalamic paraventricular nucleus (PVN) as a consequence of brain serotonin depletion. Strikingly, these defects are rescued following reestablishment of brain 5-HT signaling via administration of the serotonin precursor 5-hydroxytryptophan (5-HTP). Finally, 3D reconstruction of serotonergic fibers reveals that changes in serotonin homeostasis affect axonal branching complexity. These data demonstrate that maintaining proper serotonin homeostasis in the adult brain is crucial to preserve the correct serotonergic axonal wiring.

## Visual Abstract

**Figure:**
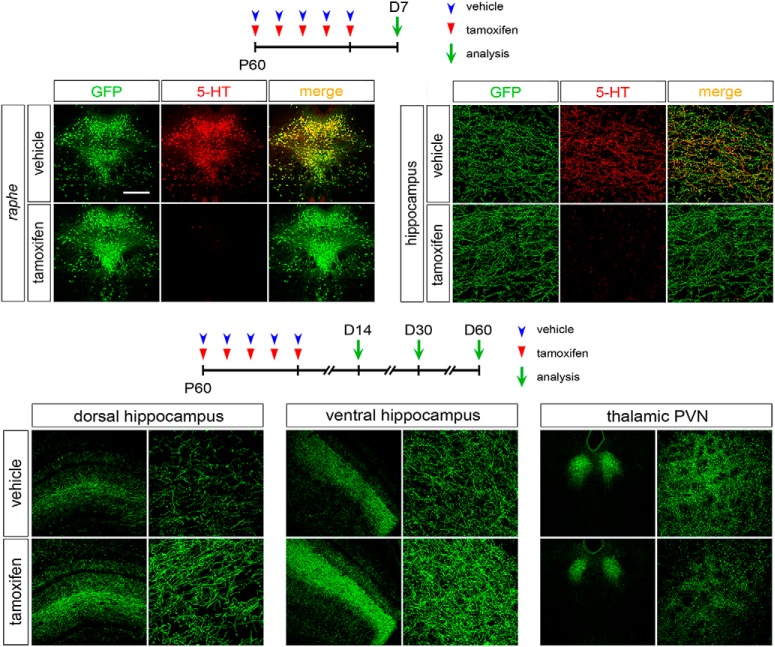

## Significance Statement

Alterations in brain serotonin (5-HT) levels during development can interfere with neuronal circuitry establishment and contribute to behavioral disturbances in adult life. It remains enigmatic whether wiring patterns can be reshaped by fluctuations of 5-HT content in the adult brain. In this study, we show for the first time that the adult serotonergic circuitry is susceptible to perturbations of serotonin homeostasis. These results provide evidence of life-long requirement of proper 5-HT levels to preserve the serotonergic axonal wiring.

## Introduction

Serotonin (5-hydroxytryptamnine, 5-HT) is a monoaminergic neuromodulator that orchestrates a broad array of processes from autonomic, cognitive and behavioral functions to the modulation of specific morphogenetic events during neurodevelopment (Gaspar et al., 2003; Ruhé et al., 2007; Alenina et al., 2009; Daubert and Condron 2010; Asan et al., 2013; Migliarini et al., 2013; Suri et al., 2015).

Neurons that synthetize serotonin are specified early during embryogenesis in the mouse hindbrain, where they cluster to form B1 to B9 *raphe* nuclei. Despite the fact that serotonergic neurons are relatively few in number (25,000 in mice; Ishimura et al., 1988), long-range projections and highly collateralized axons allow them to establish diffuse axonal trajectories to innervate the whole CNS (Gagnon and Parent 2014). By the end of gestation, serotonergic axon terminals have reached all the brain regions that will receive serotonergic innervation, and during the first three postnatal weeks, they actively sprout to profusely innervate their targets (Lidov and Molliver, 1982; Wallace and Lauder, 1983).

Maternal and placental sources of serotonin are essential for the development of the fetus (Côté et al., 2007; Bonnin et al., 2011; Goeden et al., 2016). This evidence, along with the early presence of serotonergic receptors in the developing brain, suggests that 5-HT plays a role in the orchestration of specific neurodevelopmental events (Lauder, 1993; Gaspar et al., 2003). Indeed, it has been extensively reported that an appropriate serotonergic neurotransmission is essential for the correct development of the CNS. Examples emerged from studies on animal models in which a perturbation of 5-HT homeostasis was obtained by inactivating genes coding for the serotonin transporter (SERT) or for the monoamine oxidase A. In these mice, an excess of brain serotonin during early development resulted in the disruption of topographically organized whisker-barrel fields in the somatosensory cortex (Cases et al., 1996; Persico et al., 2001) and in an abnormal migration of cortical neurons (Riccio et al., 2009; Murthy et al., 2014). Interesting insights into the role of serotonin during neurodevelopment derived also from the generation of *tryptophan hydroxylase 2* knockout (*Tph2* KO) mice, in which the rate-limiting enzyme for serotonin biosynthesis in the CNS was inactivated (Savelieva et al., 2008; Alenina et al., 2009; Gutknecht et al., 2012; Migliarini et al., 2013). Although these mice failed to show gross brain malformations, a broad number of alterations was identified, suggesting that serotonergic signaling is essential for the fine-tuning modulation of neurodevelopmental events. Indeed, *Tph2* KO mice showed physiologic modifications, altered behavior, impaired adult neurogenesis in response to running, and severe alterations in serotonergic circuitry development (Klempin et al., 2013; Migliarini et al., 2013; Mosienko et al., 2015).

Regarding the latter aspect, an important question that remains to be addressed is whether serotonin, besides its trophic role during development, is also required in the adult brain for the maintenance of serotonergic circuitry. Here, we used a *Tph2^fl^°^x^* conditional KO (cKO) mouse line to test whether severe unbalances in brain 5-HT neurotransmission during adulthood could affect the organization of serotonergic circuitry. We show that the disruption of 5-HT synthesis in adult mice deeply affects serotonergic innervation of rostral brain targets with a region-specific effect, and that a close-to-normal innervation pattern is reestablished when brain serotonin signaling is restored by chronic administration of the serotonin precursor 5-hydroxytryptophan (5-HTP). On the whole, these results prove that an appropriate serotonin homeostasis is crucial for the maintenance of serotonergic circuitry during adult stages of life.

## Materials and Methods

### Animals

Animals were maintained on artificial 12/12 h light/dark cycle at constant temperature of 22 ± 1°C and were housed in standard Plexiglas cages with food and water *ad libitum*. All experimental protocols were conducted in accordance with the Ethic Committee of the University of Pisa and approved by the Veterinary Department of the Italian Ministry of Health.

To allow detection of serotonergic fibers independently of 5-HT immunoreactivity and to abrogate serotonin synthesis in a time-controlled manner, *Tph2^GFP/fl^°^x^::CMVCreER^T^* mice were generated (Feil et al., 1996, RRID: MGI_2387570; Migliarini et al., 2013, RRID: MGI_5442773; Pelosi et al., 2015, RRID: MGI_5823194). Animals were on C57BL/6 background obtained by backcrossing with C57BL/6 mice for at least nine generations.

All animals used in the experiments were males. Mice from the same litter were treated with either drugs or vehicle/saline and samples were processed in parallel to minimize treatment-independent variability.

### Pharmacological treatments

*Tph2^GFP/fl^°^x^::CMVCreER^T^* mice were injected intraperitoneally once a day starting at postnatal day 60 (P60) for five consecutive days with 75 mg/kg body weight tamoxifen (TM, Sigma-Aldrich) dissolved in sunflower oil/ethanol (1:9) as previously described (Pelosi et al., 2015). In parallel, littermates of the same genotype were injected with sunflower oil/ethanol (1:9; vehicle) and were used as controls.

Mice were chronically administered with 5-HTP for 30 consecutive days starting form three months of age for *Tph2^GFP^* mutant mice, and 30 days after the last TM-injection for TM-treated *Tph2^GFP/fl^°^x^::CMVCreER^T^* mice. For chronic 5-HTP treatment, mice were intraperitoneally injected twice a day (9 A.M. to 7 P.M.) with 5-HTP (Sigma-Aldrich; 20 mg/kg) dissolved in sodium chloride 0.9% (saline) for 30 consecutive days. In parallel, to prevent an excessive decline in 5-HT concentration few hours after intraperitoneal injections (Mosienko et al., 2015), animals received a supplementation of 5-HTP dissolved in drinking water. 5-HTP concentration in drinking water was calculated to reach the final dose of 300 mg/kg/d, taking into account the weight of the animal and the average amount of water consumed daily. Control animals of the same litter were injected with saline and received water not supplemented with 5-HTP.

### Immunohistochemistry and *in situ* hybridization (ISH)

Immunohistochemistry and ISH were performed following standard protocols (Pelosi et al., 2014). Briefly, for immunohistochemistry, animals were deeply anesthetized with intraperitoneal injection of Avertin (1.25% solution of 2, 2, 2-tribromoethanol, Sigma-Aldrich) at 0.02 ml/g body weight and perfused transcardially with PBS followed by 4% paraformaldehyde (PFA). Brains were dissected out and postfixed overnight (o/n) at 4°C in 4% PFA before being sectioned with a vibratome (Leica Microsystem) to obtain 50-μm-thick coronal sections. Free-floating sections were cryoprotected and a one-in-three series of sections for each brain was incubated with primary antibody.

To detect 5-HT and GFP colocalization in serotonergic neurons and fibers, sections were incubated o/n at 4°C with a solution of rabbit anti-5-HT (1:500, Sigma-Aldrich, RRID: AB_477522) and chicken anti-GFP (1:1000, Abcam, RRID: AB_300798) primary antibodies in PBS containing 5% heat-inactivated lamb serum and 0.5% Triton X-100. Section were then incubated o/n in the same solution containing Rhodamine Red-X goat anti-rabbit IgG 1:500 (Thermo Fisher Scientific, RRID: AB_10374436) and Alexa Fluor 488 goat anti-chicken IgG 1:200 (Thermo Fisher Scientific, RRID: AB_2534096) secondary antibodies. For the analysis of serotonergic fibers, brain sections were immunostained for GFP using a rabbit anti-GFP primary antibody (1:2000, Invitrogen, RRID: AB_221570). To avoid the interference of endogenous GFP fluorescence in the relative optical density (ROD) quantification, a Rhodamine Red-X-conjugated goat anti-rabbit IgG antibody was used (1:500, Thermo Fisher Scientific, RRID: AB_10374436). To determine background fluorescence adjacent sections were processed omitting the primary antibody. Cell nuclei were counterstained with DAPI 0.5 mg/ml (Sigma-Aldrich).

For SERT labeling, sections were first incubated for 30 min in 0.05% hydrogen peroxide in PBS and 0.03% Triton X-100 to block endogenous peroxidase activity, and subsequently incubated for 36 h with a rabbit anti-SERT antibody (1:500, Millipore, RRID: AB_612176) in PBS containing 10% heat-inactivated lamb serum and 0.3% Triton X-100. To achieve signal amplification the avidin-biotin-HRP conjugate solution (VECTASTAIN Elite ABC kit, Vector Labs, RRID: AB_2336810) was used, following the manufacturer’s instructions. Staining was obtained by a 10-min incubation with 3-30 diaminobenzidine tetrahydrochloride (Sigma-Aldrich). For ISH, brains were dissected out, embedded in TISSUE Tek (Sakura), frozen on dry ice, and stored at -80°C. Coronal 14-μm-thick sections were cut using a cryostat and ISH was performed accordingly to standardized protocols using a ^35^S-labeled *BDNF* antisense RNA probe previously described (Migliarini et al., 2013). Sections were then exposed to Biomax MR x-ray films (Kodak) for 2 d.

### Western blot analysis

For Western blot analysis, mice were killed by decapitation, and the hippocampus was dissected out, frozen in liquid nitrogen, and stored at −80°C. Hippocampal samples were sonicated in a lysis buffer (320 mM sucrose, 50 mM Tris HCl, pH 7.5, 50 mM NaCl, 1% Triton X-100, 5 mM β-glycerol phosphate, 1 mM Na_3_VO_4_, 5 mM NaF, protease inhibitor cocktail) and, after a 30-min incubation in ice and 10-min spinning at 12,000 × *g*, the supernatant was collected. After protein determination by Bio-Rad Protein Assay kit (Bio-Rad), equal amounts of total proteins (30 μg) for each hippocampal sample were loaded onto both 10% and 15% polyacrylamide gels to determine BDNF and tyrosine receptor kinase B (TrkB) levels, respectively. Proteins were separated by SDS-PAGE and transferred overnight to membranes (Immobilon PVDF Membrane, Millipore). Then, membranes were immunoblotted overnight using selective antibodies against BDNF (1:500, Santa Cruz Biotechnology, RRID: AB_630940) and TrkB (1:1000, Santa Cruz Biotechnology, RRID: AB_2155274). Blots were then incubated with appropriate HRP-conjugated secondary antibody and target proteins were visualized by ECL detection (GE Health Care), followed by quantification through Quantity One software (Bio-Rad, RRID: SCR_014280). All optical density values of BDNF and TrkB were normalized using an antibody against GAPDH (1:5000, Santa Cruz Biotechnology, RRID: AB_627679) and α-tubulin (1:50,000, Sigma-Aldrich, RRID: AB_477593), respectively.

### Images analysis and quantification

Analysis of serotonergic fiber density was conducted on coronal brain sections immunostained for GFP. All the pictures were randomized before signal quantification, and the operator who performed the analysis was blinded to genotype and treatment.

The density of serotonergic fibers was manually quantified by ROD measurements following the procedure previously described by Migliarini et al. (2013). Images were taken with Eclipse Ti microscope (Nikon) using 10× or 4× objectives depending on the brain structure under analysis and constant acquisition parameters, and using both rhodamine and DAPI filter. Images were then converted to gray-scale (8-bit) and the optical density (OD) was quantified within the area of interest in both hippocampus and thalamic paraventricular nucleus (PVN) taking advantage of the DAPI staining using ImageJ software (RRID: SCR_003070). To calculate ROD values, the background fluorescence was subtracted from the measured OD. Background fluorescence was assessed by measuring the OD on control adjacent sections processed omitting the primary antibody.

To measure the intensity of GFP immunosignal within serotonergic neurons, images were acquired with a Nikon-A1 confocal microscope using a 10× objective and constant acquisition parameters. Images were composed of 17 z-stacks obtained at 0.3-μm step size (voxel size of 1.24, 1.24, and 1 μm/pixel in *x*-, *y*-, and *z*-dimensions, respectively). For each animal, three representative images of the median and dorsal *raphe* nuclei were acquired and the intensity of GFP fluorescence within 5-HT neurons was measured using the Surface tool of the Imaris Bitplane software (version 7.2.3, RRID: SCR_007370).

For the quantification of *BDNF* mRNA expression in the hippocampus, autoradiography films resulting from radioactive ISH were scanned at a resolution of 3200 dpi. The OD values were evaluated using the ImageJ software in the pyramidal cell layer of Ammon’s horn (CA) and in the granule cell layer of the dentate gyrus (DG) of the hippocampus. To obtain the ROD value, for each analyzed section the background OD was determined in structures of the same section devoid of signal. At least five sections for each animal were quantified.

For 3D morphologic analysis of serotonergic fibers, high-magnification confocal images were acquired using a Nikon-A1 confocal microscope. Acquisitions were performed using a 60× objective at a digital zoom of 3.0 and were composed of 60 z-stacks obtained at 0.3 μm step size (voxel size of 0.07, 0.07, and 0,3 μm/pixel in *x*-, *y*-, and *z*-dimensions, respectively). For each animal, three consecutive sections from a one-in-three series along the antero-posterior axis were analyzed performing the acquisitions at the level of the stratum lacunosum moleculare using the same dorso-ventral and lateral coordinates. Accordingly to the procedure described by Scott et al. (2013), the semiautomatic fiber tracking software Imaris Bitplane (version 7.2.3, RRID: SCR_007366) was used to generate a 3D map of serotonin axons. Computer-based quantifications of morphologic parameters such as axon length (AL), axon mean diameter, number of axon branch points and tortuosity index were automatically performed by Imaris software on the reconstructed fibers.

### Statistical analysis

Tests of significance for two-sample comparisons were performed using two-tailed Wilcoxon singed rank exact test for paired data or two-tailed Student’s *t* test. For three-sample comparison, we used Friedman test followed by two-tailed Wilcoxon signed rank exact test. For 3D reconstruction of GFP-positive fibers, Friedman test followed by one-tailed Wilcoxon signed rank exact test was used. Bonferroni’s correction was applied in case of multiple comparisons; *p* < 0.05 was considered statistically significant.

## Results

### Depletion of serotonin in the adult brain results in the alteration of serotonergic innervation density

**Figure 1.**
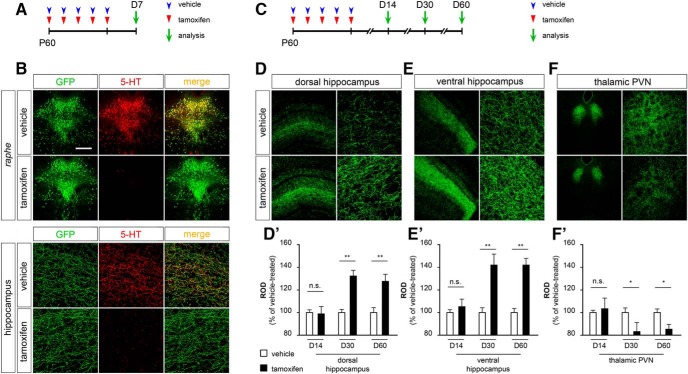
5-HT depletion in adult mice affects the density of serotonergic fibers that innervate the hippocampus and the thalamic PVN. ***A***, Experimental timeline of TM treatment in *Tph2^GFP/fl^°^x^::CMVCreER^T^* mice. ***B***, Representative images of *raphe* nuclei (top panel) and hippocampus (bottom panel) highlighting the depletion of serotonin within serotonergic cell bodies and fibers, respectively. ***C***, Experimental timeline of serotonergic fiber density analysis. ***D***–***F***, Representative coronal sections immunostained for GFP showing the distribution of serotonergic fibers in the dorsal hippocampus, ventral hippocampus, and in the thalamic PVN of vehicle- and TM-treated mice at D30. High-magnification images are shown on the right column of each panel. ***D’***–***F’***, Quantification of serotonergic fiber density in the hippocampus and in the thalamic PVN of TM- and vehicle-treated mice. Quantitative analyses were performed by means of ROD measurements at D14 (*n* = 4 for each treatment), D30 (*n* = 7 for the hippocampus; *n* = 5 for the thalamic PVN), or D60 (*n* = 8 for the hippocampus; *n* = 6 for the thalamic PVN). Data are presented as percentage increase/decrease of ROD values of TM-treated mice as compared with vehicle-treated controls ± SEM. Wilcoxon singed rank test for paired data were used as test of significance. **p < 0.01; *p < 0.05; n.s., not significant, *p* > 0.05. Scale bar, 350 μm (***B***, top panels; ***D–F***, left panels), 60 μm (***B***, bottom panels), 30 μm (***D–F***, right panels).

To address the consequences of serotonin depletion in adulthood, we took advantage of the combination of two mouse lines. We used the *Tph2^fl^°^x^* inducible KO allele, in which *Tph2* exon 3 has been floxed allowing the abrogation of serotonin synthesis on time-specific Cre recombinase activity following TM administration (Pelosi et al., 2015). We also took advantage of the *Tph2::*e*GFP* knockin mouse line (defined *Tph2^GFP^* from now on), in which the substitution of the *Tph2* gene with the *GFP* cDNA results in both the disruption of *Tph2* expression and the expression of the fluorescent reporter mirroring the presence of serotonin in both somata and projections of serotonergic neurons in developing and adult animals (Migliarini et al., 2013). By intercrossing the two lines, we obtained *Tph2^GFP/fl^°^x^* trans-heterozygous mice, harboring an inactive *Tph2^GFP^* knockin allele and a *Tph2^fl^°^x^* allele that can be inactivated on Cre-mediated somatic recombination. *Tph2^GFP/fl^°^x^* trans-heterozygous mice were born at normal Mendelian ratios and lived a normal lifespan without showing any obvious defect. To conditionally ablate serotonin synthesis in adult animals, we then intercrossed *Tph2^GFP/fl^°^x^* mice to the *CMVCreER^T^* mouse line to obtain *Tph2^GFP/fl^°^x^::CMVCreER^T^* mice, in which *Cre* recombinase transcription is driven by a cytomegalovirus promoter and its site-specific DNA recombination activity is conditionally activated on administration of TM (Feil et al., 1996). Adult *Tph2^GFP/fl^°^x^::CMVCreER^T^* animals were treated at P60 with TM once a day for five consecutive days to deplete serotonin synthesis as previously described (Pelosi et al., 2015). TM-treated *Tph2^GFP/fl^°^x^::CMVCreER^T^* mice, analyzed one week after the end of TM treatment, showed an almost complete loss of serotonin immunoreactivity in the *raphe* nuclei as previously shown (Pelosi et al., 2015) as well as in serotonergic terminals reaching the rostral brain, as compared with vehicle-treated control mice (Fig. 1*A*,*B*).

**Figure 2.**
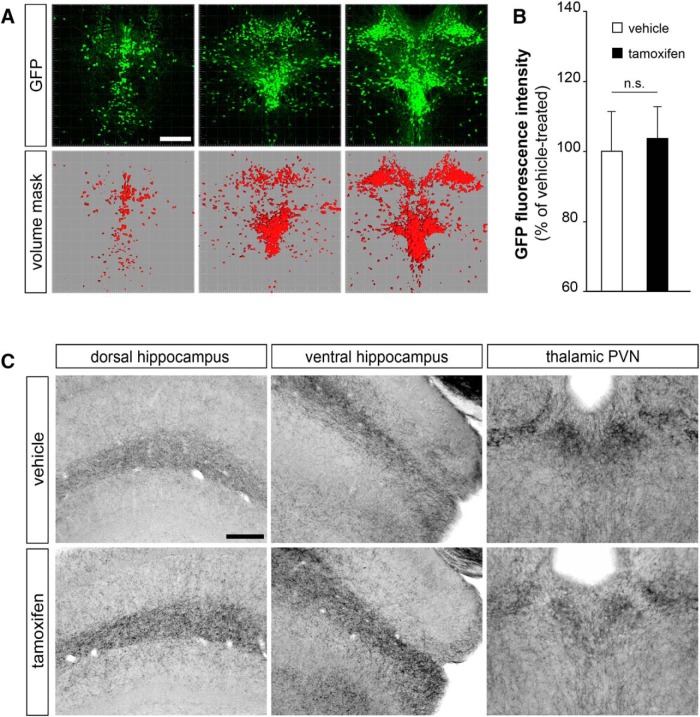
Alterations in the density of serotonergic fibers cannot be ascribed to changes in GFP expression in TM- versus vehicle-treated mice. ***A***, Representative images showing the levels along the antero-posterior axis where GFP expression was analyzed (top row) and the 3D masks used to delimit the region in which the intensity of GFP immunosignal was measured (bottom row). ***B***, Quantification of the intensity of GFP immunosignal in serotonergic neurons of TM- and vehicle-treated mice analyzed 30 days after the end of the treatment (*n* = 7 for each treatment). Analysis was performed using the Surface tool of the Imaris Bitplane software. ***C***, Representative coronal sections immunostained for SERT showing the distribution of SERT-positive serotonergic fibers in the dorsal hippocampus, ventral hippocampus, and in the PVN of vehicle- and TM-treated mice analyzed 30 days after the end of the treatment. Data were analyzed using Wilcoxon signed rank for paired data. n.s., not significant, *p* > 0.05. Scale bar, 400 μm (***A***), 200 μm (***C***, dorsal hippocampus and ventral hippocampus), and 150 μm (***C***, PVN).

As lack of central serotonin in *Tph2^GFP^* mutants has been shown to affect serotonergic fiber sprouting in the hippocampus, the nucleus accumbens, the suprachiasmatic nucleus, and the thalamic PVN with a region-specific effect (Migliarini et al., 2013), we asked whether the conditional removal of this monoamine in adulthood could result in similar alterations. We thus compared the organization of GFP-positive serotonergic fibers in TM- versus vehicle-treated *Tph2^GFP/fl^°^x^::CMVCreER^T^* animals, 14, 30, or 60 days after the last TM injection (D14, D30, and D60, respectively; Fig. 1*C*), and we focused our analysis on two brain regions such as the hippocampus and the thalamic PVN that showed pronounced and opposite alterations in *Tph2^GFP^* mutant mice (Migliarini et al., 2013). Analysis of ROD at D14 showed no significant changes in serotonin innervation between the two treatment groups in both dorsal and ventral hippocampus as well as in thalamic PVN (Fig. 1*D’–F’*). Strikingly, at D30, GFP-immunoreactive fiber density appeared to be increased in the hippocampus of TM-treated mice as compared with vehicle-treated matched controls (Fig. 1*D*,*E*). Quantitative analysis by means of ROD measurements showed a 32% significant increase in the dorsal hippocampus and 42% in the ventral part (*p* < 0.01; Fig. 1*D’*,*E’*). Conversely, the extension and density of immunoreactive serotonergic fibers appeared reduced in the thalamic PVN of D30 TM-treated animals, as confirmed by ROD analysis (−18%, *p* < 0.05; Fig. 1*F*,*F’*). To assess whether the observed alterations in ROD values could be linked to changes in GFP expression due to a different regulation of the *Tph2* promoter following 5-HT depletion, we measured the intensity of GFP immunofluorescence within serotonergic neurons in the *raphe* nuclei of both TM- and vehicle-treated mice using the Surface tool of the Imaris Bitplane software (Fig. 2*A*). Our analysis revealed no significant differences in the intensity of GFP fluorescence between TM-treated animals and controls (Fig. 2*B*). We also used SERT immunostaining as an additional assay to visualize serotonergic fibers. Results confirmed the presence of abnormalities in the density of serotonergic innervation within the hippocampus and the thalamic PVN in line with the data obtained with GFP immunoreactivity analysis (Fig. 2*C*).

Analysis of GFP-labeled serotonergic fibers at D60 confirmed the altered innervation in animals conditionally depleted of brain serotonin, showing a serotonergic hyperinnervation in both dorsal and ventral hippocampus (+28% and +41%, respectively, *p* < 0.01; Fig. 1*D’*,*E* *’*), and a reduction of fiber density in thalamic PVN (−15%, *p* < 0.05; Fig. 1*F’* ). Moreover, in line with these findings, TM-treated mice also showed an increased density of serotonergic fibers in the nucleus accumbens and a reduction at the level of the suprachiasmatic nucleus (data not shown), mirroring what has been reported in *Tph2^GFP^* mutants (Migliarini et al., 2013).

**Figure 3.**
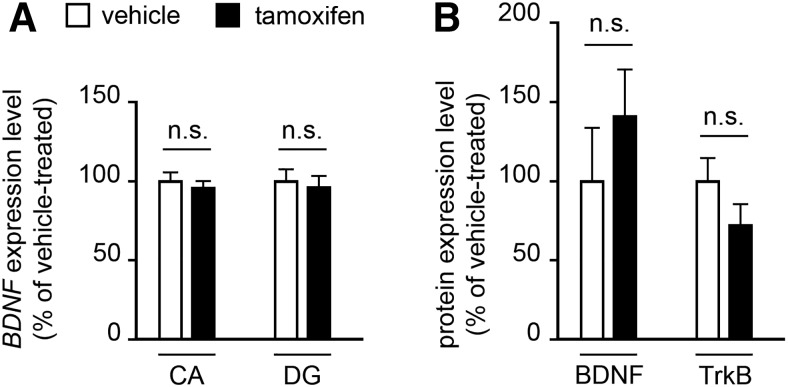
Serotonin depletion in TM-treated mice does not affect the hippocampal expression of BDNF and TrkB. ***A***, Quantification of *BDNF* mRNA expression in the hippocampus of TM- and vehicle-treated mice 30 days after the end of the treatment. The quantification was performed by densitometric analysis of autoradiograms resulting from radioactive ISH experiments (*n* = 4 for each treatment). ***B***, BDNF and TrkB protein expression level evaluated by Western blot analysis in the hippocampus of TM- and vehicle-treated mice (*n* = 3 TM-treated, *n* = 4 vehicle-treated mice) 30 days after the end of the treatment. GADPH and α-tubulin were used to normalize for variations on loading and transfer. Data are expressed as percentage of control (mean ± SEM). Student’s *t* test was used as test of significance. CA, Ammon’s horn; DG, dentate gyrus; n.s., not significant, *p* > 0.05.

To investigate whether the increased density of serotonergic fibers in the hippocampus could be linked to an increase in the hippocampal brain-derived neurotrophic factor (BDNF) regulation that is known to be influenced by serotonin signaling (Hill et al., 2011; Migliarini et al., 2013; Homberg et al., 2014; Kronenberg et al., 2016), we analyzed *BDNF* mRNA expression levels performing ISH using ^35^S-radiolabeled antisense *BDNF* probe on brain coronal sections of both TM- and vehicle-treated *Tph2^GFP/fl^°^x^::CMVCreER^T^* mice at D30. Densitometric analysis of autoradiographic signal revealed that serotonin depletion did not impact *BDNF* mRNA transcriptional regulation (Fig. 3*A*). In an additional set of experiments, we used Western blot analysis to assess the levels of BDNF and its receptor, TrkB. We found no significant correlation between either BDNF or TrkB protein level and serotonin depletion during adulthood (Fig. 3*B*).

### Reestablishing serotonin signaling rescues serotonergic innervation defects in 5-HT-depleted mice

**Figure 4.**
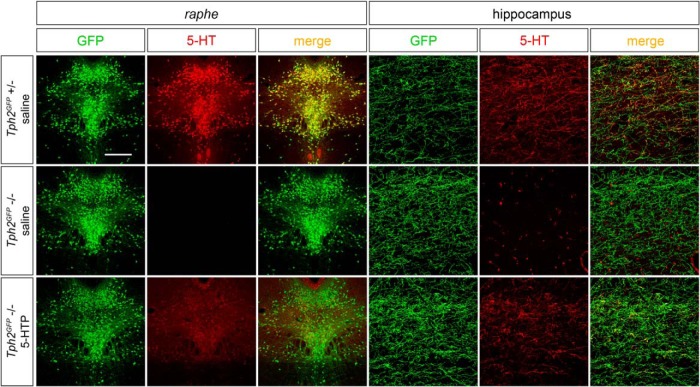
5-HTP treatment restores serotonin immunoreactivity in the brain of *Tph2^GFP^* mutants. Representative images highlighting the reestablishment of 5-HT immunoreactivity within serotonergic neurons and fibers of *Tph2^GFP^* mutant mice following 24 h of 5-HTP treatment. *Tph2^GFP^* +/− animals treated with saline were used as controls. Scale bar, 350 μm (left panels) and 60 μm (right panels).

We next explored the possibility that the serotonergic innervation defects observed in TM-treated *Tph2^GFP/fl^°^x^::CMVCreER^T^* animals could be reverted by restoring 5-HT signaling via administration of the serotonin precursor 5-HTP. First, we set up a protocol of 5-HTP administration to assess the efficiency of 5-HTP treatment in bypassing the rate-limiting step of serotonin synthesis in *Tph2*-deficient animals. To this aim, as in *Tph2^fl^°^x^* line a residual level of serotonin is maintained (Pelosi et al., 2015), we used *Tph2^GFP^* mutant mice that are devoid of brain serotonin (Migliarini et al., 2013). We found that one-day 5-HTP treatment combining two 20 mg/kg intraperitoneal injections and 300 mg/kg in drinking water was sufficient to readily detect serotonergic immunoreactivity in the *raphe* region as well as in serotonergic terminals of *Tph2^GFP^* mutant mice as compared with saline-treated ones (Fig. 4).

**Figure 5.**
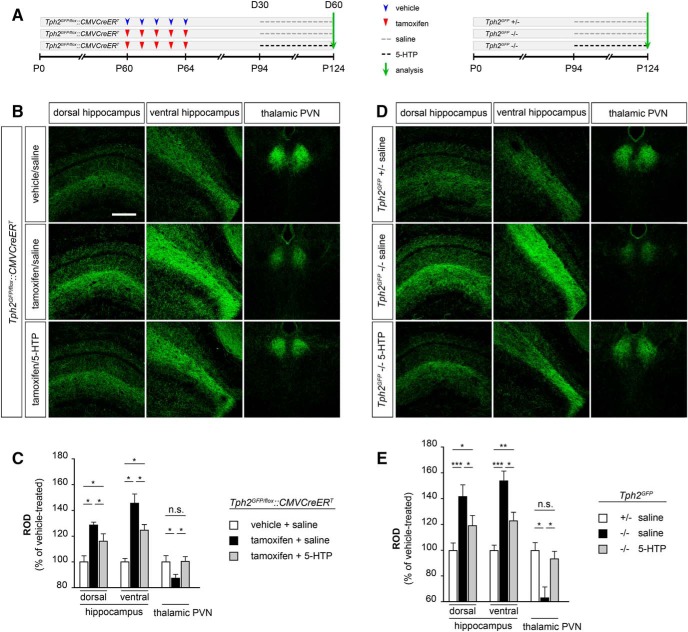
Reestablishing serotonin signaling in *Tph2*-deficient mice impacts on serotonergic fiber density and compensate alterations induced by 5-HT depletion. ***A***, Experimental design of 5-HTP administration in TM-treated *Tph2^GFP/fl^°^x^::CMVCreER^T^* mice (left) and in *Tph2^GFP^* mutant mice (right). ***B***, ***D***, Representative confocal images illustrating the distribution and density of serotonergic fibers in the hippocampus and in the thalamic PVN. ***C***, ***E***, Quantification by ROD measurements of serotonergic fiber density in the hippocampus and the thalamic PVN. Data are presented as percentage increase/decrease of ROD values as compared with vehicle/saline-treated *Tph2^GFP/fl^°^x^::CMVCreER^T^* controls (*n* = 7 for treatment) or to saline-treated *Tph2^GFP^* +/− mice (*n* = 6 for treatment) ± SEM. Data were analyzed using the Friedman test followed by the Wilcoxon signed rank test and the Bonferroni’s correction was applied. ***p < 0.001; **p < 0.01; *p < 0.05; n.s., not significant, *p* > 0.05. Scale bar, 350 μm.

We then tested whether the reestablishment of serotonin signaling could rescue the alterations of serotonergic innervation induced in *Tph2^GFP/fl^°^x^::CMVCreER^T^* animals following TM treatment. *Tph2^GFP/fl^°^x^::CMVCreER^T^* mice that had received the 5-days TM treatment starting at P60 to deplete serotonin synthesis were then administered at D30 (namely P94) with either saline (i.e. TM/saline) or 5-HTP (i.e. TM/5-HTP) for the following 30 days (Fig. 5*A*). Immunohistochemical analyses confirmed in both dorsal and ventral hippocampus as well as in the thalamic PVN of *Tph2^GFP/fl^°^x^::CMVCreER^T^* TM-treated mice that received saline the presence of an aberrant serotonergic innervation in line with that described in Figure 1 (Fig. 5*B*). Quantitative analysis by ROD measurements showed a 28% (*p* < 0.05) and 45% (*p* < 0.05) increase of serotonergic fiber density in dorsal and ventral hippocampus, and a 13% (*p* < 0.05) decrease in the thalamic PVN as compared with control animals, respectively (Fig. 5*C*). Strikingly, GFP-positive serotonergic fibers in *Tph2^GFP/fl^°^x^::CMVCreER^T^* TM-treated animals appeared less packed in both dorsal and ventral hippocampus following 30 days of 5-HTP treatment as compared with their saline-treated controls (Fig. 5*B*). In parallel, we observed a noticeable increase in the complexity of GFP fibers in the thalamic PVN of 5-HTP-treated mice as compared with control animals (Fig. 5*B*). Consistently, ROD measurements highlighted a significant reduction in serotonergic terminal density of TM/5-HTP mice in the dorsal (−13%, *p* < 0.05; Fig. 5*C*) and ventral (−21%, *p* < 0.05; Fig. 5*C*) hippocampus and a +13% (*p* < 0.05) increase in the thalamic PVN, as compared with TM/saline controls (Fig. 5*C*).

In light of these results, we next asked whether the reestablishment of the serotonergic signaling could have an impact on the 5-HT innervation of animals that are life-long depleted in brain 5-HT such as the *Tph2^GFP^* mutant mice. To address this question we submitted 3 months old *Tph2^GFP^* mutant mice to our 5-HTP administration protocol. Interestingly, following 30 days of 5-HTP treatment, we observed a reduction of the serotonergic innervation abnormalities previously described in *Tph2^GFP^* mutant mice (Fig. 5*D*). Analysis in both dorsal and ventral hippocampus highlighted a 22% (*p* < 0.05) and 31% (*p* < 0.05) reduction of fiber density in 5-HTP-treated *Tph2^GFP^* mutants as compared with saline-treated controls (Fig. 5*E*). In line, reestablishment of serotonin signaling was sufficient to restore a proper serotonergic innervation in the thalamic PVN of 5-HTP-treated *Tph2^GFP^* −/− mice as compared with saline-treated mutant animals (+32%, *p* < 0.05; Fig. 5*D*,*E*).

Taken together, our data suggest that the serotonergic system retains a lifelong plasticity allowing it to respond to transient alterations in brain serotonin homeostasis and resulting in changes of fiber density in the target areas. Moreover, results showed that innervation defects observed in 5-HT-depleted mice can be significantly rescued by 5-HTP treatment capable to reestablish serotonin levels.

### The depletion/reestablishment of 5-HT signaling in adult brains affects the sprouting of serotonergic fibers

Since we demonstrated that 5-HT signaling abrogation/reestablishment deeply affects the density of 5-HT fibers innervating the hippocampus, we next wanted to decipher which morphologic rearrangements in the architecture of 5-HT axons are responsible for the observed alterations in both *Tph2^GFP/fl^°^x^::CMVCreER^T^* and *Tph2^GFP^* animals. We hypothesized that both an enlargement in axon diameter, and/or an active axon sprouting resulting in an increased complexity of serotonergic innervation could account for the higher density of 5-HT fibers we have observed, while both diminished axon diameter and/or branching regression could explain the fiber density reduction.

**Figure 6.**
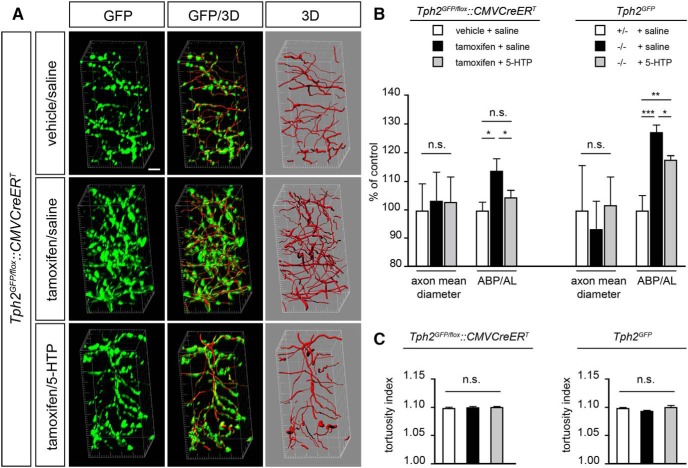
Computer-based mapping of serotonergic fibers and quantitative analysis of axon morphology. ***A***, Representative high-magnification confocal acquisition in oblique view of GFP-immunoreactive serotonergic fibers (green) and computer-based 3D reconstructions (red). ***B***, Graphs showing the quantification of the axon mean diameter and the ABP/AL ratio in *Tph2^GFP/fl^°^x^::CMVCreER^T^* (*n* = 5 for treatment) and *Tph2^GFP^* (*n* = 5 for treatment). Data were normalized to vehicle/saline-treated *Tph2^GFP/fl^°^x^::CMVCreER^T^* controls and saline-injected *Tph2^GFP^* +/− controls, respectively, and presented as mean percentage increase of controls ± SEM. ***C***, Graphs showing the average tortuosity index (defined as the length of the axon divided by the Euclidean distance between the ends or branch points) of serotonergic fibers under the different experimental conditions. Data are presented as the mean ± SEM. Analysis of significance was performed using the Friedman test, followed by the Wilcoxon signed rank test. ***p < 0.001; **p < 0.01; *p < 0.05; n.s., not significant, *p* > 0.05. Scale bar, 4 μm.

To test this hypothesis we analyzed high-magnification confocal image acquisitions of GFP-labeled 5-HT fibers in the stratum lacunosum moleculare of the dorsal hippocampus. Imaris software was used to generate 3D reconstructions of GFP-immunoreactive axons thus allowing an accurate computer-based quantitative and morphologic fiber analysis (Fig. 6*A*). Results showed no significant difference in the diameter of serotonergic fibers among the different experimental groups in either *Tph2^GFP/fl^°^x^::CMVCreER^T^* or *Tph2^GFP^* mice (Fig. 6*B*). Conversely, a significant increase of the length of 5-HT fibers and number of axon branch points was observed between *Tph2^GFP/fl^°^x^::CMVCreER^T^* vehicle/saline- and TM-saline-treated animals resulting in +14% axon branch point (ABP) versus axon length (AL) ratio (ABP/AL ratio; *p* < 0.05; Fig. 6*B*). A similar scenario was observed comparing *Tph2^GFP^* +/− and −/− saline-treated mice (+26% ABP/AL ratio; *p* < 0.05; Fig. 6*B*). Notably, 5-HTP administration in TM-treated *Tph2^GFP/fl^°^x^::CMVCreER^T^* animals promoted a decrease in the total 5-HT fiber length and the number of branching points, bringing back the ABP/AL ratio to the level of that observed in control mice (Fig. 6). In *Tph2^GFP^* −/− mice, 5-HTP treatment promoted a significant decrease of the ABP/AL ratio, although the rescue was partial (ABP/AL ratio +17% as compared with *Tph2^GFP^* +/− controls; *p* < 0.01; Fig. 6*B*). Finally, to further evaluate the consequences of changes in serotonin homeostasis on serotonergic fiber properties, we compared the tortuosity of GFP-immunoreactive fibers between control and experimental animal cohorts (Fig. 6*C*). We found that the tortuosity index of serotonergic axons is not affected by serotonin depletion/reestablishment, suggesting that their morphologic properties are retained independently of serotonin level fluctuations (Fig. 6*C*).

Overall, 3D reconstruction of serotonergic axon architecture and morphology quantification demonstrated that 5-HT signaling abrogation/reestablishment in adult mice can affect proper serotonergic fiber density impinging on axon branching complexity, but not on morphologic properties, by either promoting or inhibiting axon sprouting.

## Discussion

This study aims to assess whether an appropriate serotonergic neurotransmission during adult life is required to preserve proper serotonergic circuitry. Here, we show that depletion of brain serotonin in adult mice produces severe abnormalities in serotonergic fiber density with a region-specific effect and that these alterations can be reversed with chronic 5-HTP administration that restores 5-HT signaling.

To efficiently address the consequences of 5-HT depletion in adult brains, we analyzed the density of serotonergic fibers focusing on the hippocampus and the thalamic PVN that displayed diametrically opposite phenotypes in the *Tph2* KO mouse model as a consequence of brain 5-HT depletion (Migliarini et al., 2013). Remarkably, we report that 5-HT synthesis abrogation in adult *Tph2* cKO mice results, after four weeks, in the alteration of serotonergic fiber density as observed by the analysis of both SERT and GFP immunoreactivity. Such a phenotype mimics the abnormalities observed in mice with a life-long depletion of brain serotonin. We also show that the changes of fiber density induced by serotonin depletion can be rescued by restoring brain 5-HT signaling in *Tph2* cKO as well as in *Tph2* KO animals. These results corroborate the hypothesis that serotonin itself acts as a modulator of serotonergic fiber density, as already proposed to explain the phenotype of *Tph2^GFP^* mutants (Migliarini et al., 2013). Serotonin is known to exert a trophic autocrine activity during development and at postnatal stages when 5-HT axons sprout to innervate their targets (Lidov and Molliver, 1982). Our findings reveal that the impact of serotonin on serotonergic axon sprouting is not restricted to critical periods during the course of neurodevelopment, but it is maintained in adulthood. Altogether, the ability of serotonin to act as a regulator of serotonergic axon sprouting and the evidence that this feature is retained during adult stages of life reveal an unexpectedly high plasticity of adult serotonergic circuitry, putting forward its responsiveness to fluctuations in brain serotonin content.

Growing evidence for a structural plasticity of axons in the adult brain, defined as the ability to rearrange and sprout under specific circumstances, such as spontaneous or experience-dependent activity, has been reported in different neuronal types (De Paola et al., 2006; Stettler et al., 2006; Gogolla et al., 2007; Butz et al., 2009; Yamahachi et al., 2009; Barnes and Finnerty, 2010; Marik et al., 2010, 2013, 2014). To date, the ability of adult serotonergic axons to undergo active sprouting has been described solely in response to physical lesions of brain or spinal cord (Zhou and Azmitia, 1984; Azmitia and Whitaker-Azmitia, 1991; Zhou et al., 1995; Camand et al., 2004; Hawthorne et al., 2011; Jin et al., 2016). However, tissue lesion represents a nonphysiologic condition in which the environment is enriched with a large repertoire of molecules, which possess the ability to influence the behavior of local 5-HT fibers (Dusart et al., 1999; Camand et al., 2004; Hawthorne et al., 2011). Our findings go beyond this view showing that axonal structural plasticity represents an intrinsic property of serotonergic fibers independently of tissue damage and that plasticity of serotonergic terminals can be modulated by changes in serotonergic homeostasis. On the other hand, an intriguing question is how the manipulation of brain serotonin levels may trigger bidirectional dynamic structural rearrangements of serotonergic fibers, such as axon sprouting or reduction of axon arbor complexity. A tempting hypothesis is that, as reported for other neuronal types (De Paola et al., 2006; Stettler et al., 2006; Nishiyama et al., 2007), serotonergic fibers are highly dynamic in physiologic conditions undergoing continuous small rearrangements, such as branch elongation and retraction. When drastic changes in 5-HT brain levels occur, such as those induced by TM treatment in conditional *Tph2* KO or by chronic 5-HTP administration, the dynamic equilibrium of 5-HT axons is unbalanced toward either elongation or retraction, resulting in fiber sprouting or reduction of axon arbor complexity, respectively.

Another aspect that remains elusive is how serotonin depletion/reestablishment can produce diametrically opposite phenotypes of serotonergic innervation to specific structures, such as the hippocampus and the thalamic PVN. On one side, we can hypothesize that an intrinsic heterogeneity in the targets might be implied in the distinct response of serotonergic fibers to fluctuations of 5-HT levels. Indeed, as the distribution of serotonergic receptors and their downstream effectors is highly variable in the rostral brain (Wirth et al., 2016), the response to serotonin deregulation is the outcome of the integration of signals encoded by the specific combination of receptors expressed in each target region. On the other side, it is likely that 5-HT neurons projecting to the hippocampus and the thalamic PVN possess intrinsic differences that might contribute to the opposite effect elicited by serotonin depletion/reestablishment. Indeed, although serotonergic fibers innervating the hippocampus and the thalamic PVN manly arise from neurons of same *raphe* nucleus, namely the median *raphe* nucleus (Muzerelle et al., 2016), there is now clear evidence that a marked intrinsic heterogeneity exists even among serotonergic neurons belonging to the same anatomic domain. Indeed, distinct subtypes of 5-HT neurons that differ in their developmental origin (Jensen et al., 2008), molecular profile (Okaty et al., 2015) as well as in their electrophysiological properties and cotransmitter repertoire (Gaspar and Lillesaar, 2012; Fernandez et al., 2015) have been identified, suggesting the possibility that specific subtypes of serotonergic neurons may be specialized to mediate different biological functions. Moreover, serotonergic neurons that project to distinct forebrain regions largely belong to different serotonergic subpopulations (Fernandez et al., 2015). Based on this evidence, our observation of an opposite phenotype after serotonin depletion/reestablishment suggests that serotonergic fibers innervating the hippocampus and the thalamic PVN may arise form distinct subpopulation of median *raphe* 5-HT neurons. Overall, it is likely that the specific combination of serotonergic receptors in target areas along with the intrinsic properties of 5-HT neurons projecting to the same target contribute to the establishment of a regional specificity in the way changes in serotonin levels drive 5-HT fiber rearrangements.

It has been reported that modulating 5-HT signaling by administration of either fluoxetine or agonists of 5-HT receptors facilitates the recovery of locomotion after a spinal cord injury (SCI) in rodents (Courtine et al., 2009; Musienko et al., 2011; Scali et al., 2013). However, the precise mechanisms underlying this response are still waiting to be elucidated. In this respect, an intriguing question to address would be whether a potential role in post-SCI recovery, besides the reported action of serotonin itself, could be ascribed to serotonergic axons. In fact, because of their intrinsic capability to regrow following injury, serotonergic fibers may act as forerunners facilitating the navigation and sprouting of other types of axons through the edge of the lesion. Thus, in the future, it will be interesting to evaluate whether (1) the capability of serotonergic axons to actively sprout in response to injury and (2) the post-SCI recovery triggered by modulation of 5-HT signaling are retained in the absence of serotonin, such as in our *Tph2* cKO.

As BDNF expression has been reported to be influenced by serotonin signaling (Hill et al., 2011; Migliarini et al., 2013; Homberg et al., 2014; Kronenberg et al., 2016), we wondered whether this neurotrophin could act as a potential molecular mediator in the establishment of the observed alterations in 5-HT fiber density. However, we failed to detect any significant alteration in the hippocampal level of BDNF in our *Tph2* cKO mice, suggesting that other factors are likely involved in the response of 5-HT depletion during adult stages of life. Noteworthy, the increase of hippocampal BDNF in *Tph2* mutant mice is detectable as early as P14, indicating that early phases of postnatal development represent a temporal window in which serotonin depletion can affect BDNF expression. Conversely, it is likely that the ability to influence BDNF hippocampal levels is lost in adult brains. Accordingly, the existence of specific temporal windows during postnatal development in which 5-HT signaling produces specific effects that cannot be replicated later in life has been extensively demonstrated (Gross et al., 2002; Ansorge et al., 2004, 2008; Suri et al., 2015).

On the whole, we have shown that drastic changes in brain 5-HT content during adulthood affect, in a reversible manner, serotonergic fiber density and complexity, thus providing the evidence of a direct link between 5-HT homeostasis and structural plasticity of serotonergic axons in the adult brain. In this view, it should be taken into account that also subtle changes of brain 5-HT homeostasis induced by genetic factors, pharmacological treatments, as well as environmental conditions, might impinge on serotonergic terminal sprouting, thus affecting the proper 5-HT circuitry and, potentially, interfering with its functioning.

## Synthesis

Reviewing Editor: Alfonso Represa, INSERM Decisions are customarily a result of the Reviewing Editor and the peer reviewers coming together and discussing their recommendations until a consensus is reached. When revisions are invited, a fact-based synthesis statement explaining their decision and outlining what is needed to prepare a revision will be listed below. The following reviewer(s) agreed to reveal their identity: Jerry Silver, Patricia Gaspar.

This is a concise report convincingly demonstrating that when 5-HT transmission is reduced raphe terminal innervation is modified within 30 days. The study complements previous observation by the same team showing similar effects in constitutive Tph2-KO mice. The novelty here is to show that, contrary to what one might expect, this is not a developmental phenotype, but reflects a dynamic process also observed in the adult; Maintaining or removing 5-HT innervation in targets is controlled by the level of brain 5-HT.

Although mechanistic insights are lacking the observation is clear and well documented; the phenotype in the conditional KO mice mimics the one observed in the full-KO mice and, conversely, increasing 5-HT in the Tph2-KO mice reversed the phenotype.

All the study rests on morphological measures of GFP-immunostaining that is driven by the Tph2 promoter. Can the authors exclude the fact that the observed effect is not an up- regulation or down-regulation of the Tph2 gene that would result in a change in GFP expression, leading to a change in signal in the fiber terminals? Indeed, it seems from Figure 1B, that the intensity of GFP was increased in the dorsal raphe neurons. One way to address this question would be to look whether similar changes are observed with SERT-immunocytochemistry in the fiber terminals. Referees are aware that SERT immunostaining would provide less anatomically resolution than GFP, but it would help ratifying the conclusion of the manuscript.

Minor remarks are:

-In most articles the abbreviation “PVN” is used to refer to the paraventricular hypothalamic cell group. It would help indicating more clearly (e.g. in abstract and Figure legends) that this is the thalamic paraventricular nucleus. This could be clearly indicated in the first figure by showing on a scheme, the areas sampled for quantification.

-It should be mentioned clearly that the Tph2:eGFP mouse line is a knock-in and is therefore lacking one allele of the Tph2 gene- so recombination concerns one copy of the gene.

-Authors could discuss that the raphe neurons which undergo terminal remodeling in hippocampus and medial thalamus are those coming from the median raphe groups. Since the changes goes in both directions, this suggests heterogeneity in the median raphe, or in the targets.

The time point post tamoxifen treatment when the 5HTP treatment began should also be described at least briefly in the methods. I was wondering about this until I read it in the results section

-Is there a heightened microglial response in the areas that lose 5-HT terminals after 5 HTP treatment. At this point It is not necessary to prove that microglial pruning might be occurring but it would be interesting to just show that the microglia are activated or not.

-The Andersen et al paper is highly controversial and the authors might consider this in their citation

-There are a number of grammatical mistakes throughout. It is suggested that prior to resubmission, the paper be given to an editor in order to clarify the writing. Several mistakes are pointed out below but there are others.

• line 107 : : « thick » not “tick”

• line 147: describe better the blinding procedure-

• line 249: « did not impact BDNF » or « had no impact on BDNF » rather than « did not impact on BDNF; » 

• line 296: « compared » and not « compeer »

• brain's wiring patterns should be “wiring patterns in the brain”

• Despite serotonergic neurons are relatively small in number should be despite the fact that....

• These evidences should be This evidence

• Independently to should be independently of

• Compeer should be compared

• Allowing to should be allowing it to

• Compeer should be compared

• Allowing to should be allowing it to
